# Combinations of physiologic estrogens with xenoestrogens alter calcium and kinase responses, prolactin release, and membrane estrogen receptor trafficking in rat pituitary cells

**DOI:** 10.1186/1476-069X-9-61

**Published:** 2010-10-15

**Authors:** Yow-Jiun Jeng, Mikhail Kochukov, Cheryl S Watson

**Affiliations:** 1 Department of Biochemistry and Molecular Biology, University of Texas Medical Branch, Galveston, Texas, USA

## Abstract

**Background:**

Xenoestrogens such as alkylphenols and the structurally related plastic byproduct bisphenol A have recently been shown to act potently via nongenomic signaling pathways and the membrane version of estrogen receptor-α. Though the responses to these compounds are typically measured individually, they usually contaminate organisms that already have endogenous estrogens present. Therefore, we used quantitative medium-throughput screening assays to measure the effects of physiologic estrogens in combination with these xenoestrogens.

**Methods:**

We studied the effects of low concentrations of endogenous estrogens (estradiol, estriol, and estrone) at 10 pM (representing pre-development levels), and 1 nM (representing higher cycle-dependent and pregnancy levels) in combinations with the same levels of xenoestrogens in GH_3_/B6/F10 pituitary cells. These levels of xenoestrogens represent extremely low contamination levels. We monitored calcium entry into cells using Fura-2 fluorescence imaging of single cells. Prolactin release was measured by radio-immunoassay. Extracellular-regulated kinase (1 and 2) phospho-activations and the levels of three estrogen receptors in the cell membrane (ERα, ERβ, and GPER) were measured using a quantitative plate immunoassay of fixed cells either permeabilized or nonpermeabilized (respectively).

**Results:**

All xenoestrogens caused responses at these concentrations, and had disruptive effects on the actions of physiologic estrogens. Xenoestrogens reduced the % of cells that responded to estradiol via calcium channel opening. They also inhibited the activation (phosphorylation) of extracellular-regulated kinases at some concentrations. They either inhibited or enhanced rapid prolactin release, depending upon concentration. These latter two dose-responses were nonmonotonic, a characteristic of nongenomic estrogenic responses.

**Conclusions:**

Responses mediated by endogenous estrogens representing different life stages are vulnerable to very low concentrations of these structurally related xenoestrogens. Because of their non-classical dose-responses, they must be studied in detail to pinpoint effective concentrations and the directions of response changes.

## Background

**Figure 1:**
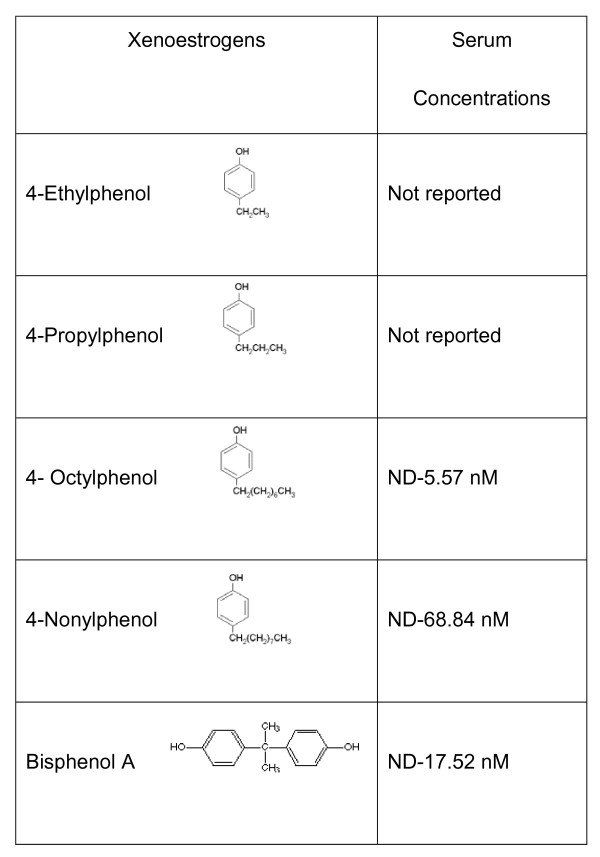
**Chemical structures and reported serum levels of xenoestrogens used in our study **[26-29]. ND (non-detectable).

Xenoestrogens are small lipophillic molecules that mimic physiological estrogens [1], and whose exposures have been linked to a variety of disease states [2-11], even when present at concentrations far below those currently allowed by federal regulations. We previously found that both physiologic estrogen metabolites and a number of environmental estrogens have potent activities via the nongenomic pathway of estrogen signaling [12-19], whereas they have been shown to be rather weak via genomic signaling mechanisms [20-25]. Therefore, inactivity in genomic assays does not necessarily predict the same via nongenomic mechanisms. A variety of xenoestrogens representing different chemical and use classes has been examined for nongenomic signaling pathway activities. Alkylphenols are of particular interest, as they represent a structurally related set of compounds that differ in the lengths of their carbon side-chain modifications at position 4 on the phenol ring, or in the case of bisphenol A (BPA) have another phenolic ring in place of the side-chain (see Figure 1 for structures and human blood levels [26-29]). The use of alkylphenols as industrial surfactants results in contamination of many of our waterways [30,31]. BPA can contaminate the environment in significant amounts by leaching from products (plastic food and water containers, dental sealants, and some cash register receipts), and as byproducts of manufacturing [32-34].

There are three candidate estrogen receptors (ERs) via which estrogenic and xenoestrogenic responses can operate. The classical ERs α and β have been described in the membrane of these and other cells [35-43]. GPER is a transmembrane ER in the GPCR family. It has been shown to mediate a wide range of responses to estrogens in different cell types [44]. Because many of the endpoints that it drives are different than those of the classical ERs α and β (though often using overlapping signaling pathways) [45], it may be a complementary estrogen signaling system in ways that we do not yet fully understand. Estrogens bind with different affinities to these different ER types [46,47], which could provide diverse physiological outcomes in unique circumstances or stages of development.

We previously compared the signaling activities of many physiological estrogens and xenoestrogens to those of estradiol (E_2_) in pituitary and neuronal cells, gaining some insight into how their chemical structure may correlate with their estrogenic activity [1,15]. These compounds signal predominantly via membrane receptors for ERα (mERα) [15,18,19], with compensatory actions sometimes occurring via mERβ or GPER when they are simultaneously present with mERα [42]. Other studies have also demonstrated potent activities of some of these xenoestrogens in pancreatic islet cells [10], neuronal cells and tissues [48,49], cells of the immune system [8,50], and a wide variety of other cell or tissue experimental systems [51]. There are also many more studies describing the activities of these compounds at much higher concentrations, which may or may not act via receptors, but have limited applicability to our concern about prevalent environmental concentrations. Lifelong exposure to E_2 _and potent synthetic estrogens is also known to be an important consideration for the development of cancers in reproductive tissues [52-55], so there is a possibility that xenoestrogens could likewise contribute to these types of disease. Xenoestrogens can cause proliferation of cells, as we demonstrated for octylphenol (OP), nonylphenol (NP), and BPA in our GH_3_/B6/F10 cell model ([15] and reports cited therein).

Few studies have examined the ability of low concentrations of xenoestrogens in combination with physiologic estrogens to alter their responses. In some cases the combined presence of these estrogens can be inferred because the studies were done in gonadally intact animals or in tissues isolated from such animals treated in vivo [8,49,56-59]. Because of the non-monotonic concentration-responses typical of nongenomic estrogenic responses [12,13,15,17,19,42,60,61], wide concentration ranges and multiple combinations must be assessed using quantitative, relatively high-throughput endpoints to determine the real profile of xenoestrogen dose-based activities. Here we extend our earlier assays of individual estrogens and xenoestrogens shown to be active in nongenomic responses, to assays of their combinatorial effects. We have developed these assays in pituitary cells, where the responses relate to a variety of cellular responses and functions, including intracellular calcium (Ca) levels; the phospho-activation of extracellular regulated kinases (ERKs 1 and 2) as representatives of the mitogen-activated protein kinase (MAPK) family often involved in cellular remodeling, proliferation or death; and the release by the pituitary of a major peptide hormone, prolactin (PRL).

**Figure 2:**
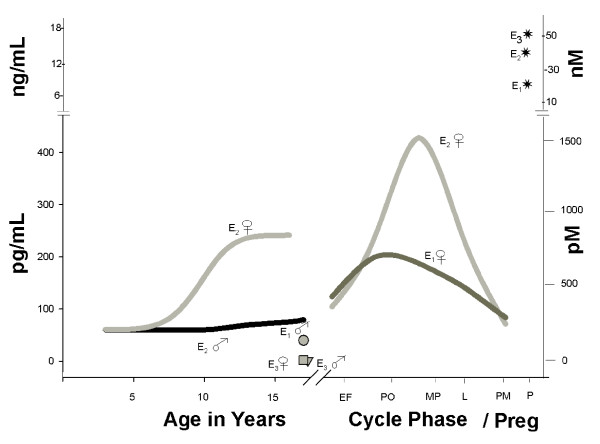
**Levels of physiologic estrogens during different life stages in females compared to males**. These data were graphed from information summarized in a textbook [62], and span concentrations over 2 orders of magnitude. Levels over time and different stages are shown by lines, and point values are shown as symbols, all labeled directly on the graph. Cycle phases are labeled on the timeline as EF (early follicular), PO (preovulatory), MP (mid cycle-peak), L (luteal), PM (post menopausal), and P (pregnant, peak levels). The levels of the estrogens estrone, estradiol, and estriol (E_1_, E_2_, and E_3_, respectively) are shown for females (♀) and males (♂)

E_2 _(in the pM to nM range) is the physiologic estrogen most often associated with female development and specific reproductive function (see Figure 2). Relevant concentrations range across more than two orders of magnitude from early developmental levels up to those that direct function during the reproductive years. However, other endogenous estrogens such as estrone (E_1_) and estriol (E_3_) are more prevalent during other life phases, and may have significant effects on tissue development, function, and disease states. E_1 _is a significant estrogenic hormone contributor in both reproductive (~0.5-10 nM) and postmenopausal (150-200 pM) women, and in men; E_3 _levels are much higher in pregnant (~10-100 nM) than in nonpregnant (<7 nM) women [62]. Low E_3 _levels in pregnancy have been associated with complications of eclampsia [63] and the incidence of Down's syndrome in offspring [64]. All three of these endogenous estrogens are also produced by aromatases in a number of non-reproductive tissues, where their effects may extend beyond reproductive functions [65]. One example is that E_3 _has protective effects against the development of arthritis in certain experimental models [66], as had been known previously for E_2_. Effects in brain, bone, the cardiovascular system, and many other tissues may be affected differentially by these three endogenous estrogenic compounds during different life stages; therefore, loss or enhancement of these effects due to interference by xenoestrogenic compounds could affect human health in a large number of tissues. Though it has recently been determined that E_1 _and E_3 _can act potently via nongenomic steroid signaling mechanisms in several tissues, including pituitary and neuronal cells [12,13,67,68], we do not know if xenoestrogens can interfere with these activities when they are present in combination, as is typical with exposures to humans and animals living in a contaminated environment.

The subcellular trafficking of signaling proteins is a critical consideration for their function, because many actions require close associations of cross-activating and signal-transducing proteins. In this regard, steroid receptors have been found to reside in the caveolar subcompartment of plasma membranes [69-71] in addition to the nuclear compartment, and the entry and exit of proteins from these compartments, and locations in between, tells part of the story of their participation in cellular regulation. We have recently found in other systems and with other estrogens that estrogenic actions can also lead to trafficking of ERs in and out of the plasma membrane [13,42]. Here we look at changes in the membrane location of ERs to better understand how xenoestrogens may alter the nongenomic actions of physiologic estrogens by re-locating their receptors to sites where nongenomic signaling partners may be less available.

Our tests of physiologic estrogens examine concentrations that might be experienced due to normal mammalian levels of the endogenous hormones at different life stages. A 10 pM concentration would approximate early developmental (infant) levels (see Figure 2), and a 100× higher 1 nM concentration of these hormones would approximate concentrations experienced during menstrual cycling or early pregnancy. We then super-imposed upon those endogenous estrogen levels a frequently encountered very low contaminant concentration range of alkylphenols and BPA (10 pM and 1 nM) to see how normal signaling and functional endpoints would be affected.

## Methods

### Materials and treatments

We purchased phenol red-free Dulbecco modified Eagle medium (DMEM, high glucose) from Mediatech (Herndon, VA); horse serum from Gibco BRL (Grand Island, NY); defined supplemented calf sera and fetal bovine sera from Hyclone (Logan, UT). Paraformaldehyde and picric acid were purchased from Fisher Scientific (Pittsburgh, PA). BPA (bisphenol A), EP (4-*n*-ethylphenol), PP (4-*n*-propylphenol), OP (4-*n*-octylphenol), and NP (4-*n*-nonylphenol), and other materials were purchased from Sigma (St. Louis, MO). The antibody (Ab) for phosphorylated (pERK) was purchased from Cell Signaling Technology (Danvers, MA), for clathrin (mouse anti-clathrin monoclonal) from ICN Biomedicals (Aurora, OH), for ERα (MC-20) from Santa Cruz Biotechnology (Santa Cruz, CA), for ERß (Clone9.88) from Sigma, and for GPER (NLS4271) from Novus (Littleton, CO). Vectastain ABC kits and biotin-conjugated secondary Abs were purchased from Vector Labs (Burlingame, CA). GH_3_/B6/F10 cells were routinely propagated in DMEM containing 12.5% horse serum, 2.5% defined supplemented calf serum, and 1.5% fetal calf serum. Cells were used between passages 10 and 20 and placed in defined or charcoal-stripped serum-containing media before assays for estrogenic activity.

### Cytosolic free calcium ([Ca^2+^]i) fluorescent imaging

**Figure 3:**
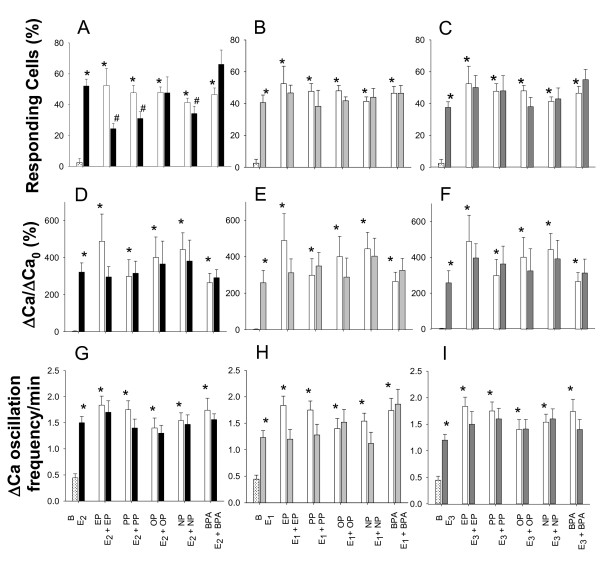
**Changes in calcium responses elicited by physiologic estrogens (PE) and xenoestrogens (XE), alone and in combination**. The level of Ca [compared to the pretreatment baseline (B) measurement for each assessed cell] was measured by Fura-2 imaging in cells treated with 10 pM E_2_, E_1_, or E_3 _or different 10 pM alkylphenols (EP, PP, OP, NP, or BPA), or physiologic estrogen and xenoestrogen combinations. (A-C) % of cells responding to estrogens; (D-F) changes in accumulated intracellular calcium levels; (G-I) changes of calcium oscillation frequency. Note - some baseline measurements (D-F) were too low to show up as bars on the graphs. * = p < 0.05 compared to control cells. # = p < 0.05 compared to E_2_, E_1_, or E_3 _alone.

Cells were plated on poly-D-Lysine-treated 35/22 mm glass bottom dishes (Willco Wells, Amsterdam, Netherlands) at a density of 100,000 cells/mm^3^. After 48-72 h of culture, the cells were loaded with the calcium-sensitive fluorescent dye Fura-2AM (Molecular Probes, Eugene, OR) at 2.5 μM (1 h, RT), washed in a physiologic solution (150 mM NaCl, 5.5 mM KCl, 1 mM MgCl_2_, 4 mM CaCl_2_, 7 mM glucose, 10 mM HEPES, pH 7.4), and incubated at RT for 1-4 h before live Ca imaging. The imaging setup included a Nikon 200E microscope with 40× SuperFluo lens and computer-controlled illumination system (Sutter Instruments, Novato, CA) equipped with a digital monochrome-cooled charge-coupled device Roper Coolsnap HQ camera (Roper Scientific, Tucson, AZ). Fluorescent emissions at 510 nm from regions corresponding to a single cell were acquired online with the MetaFluor software (Universal Imaging, Downington, PA). The signals were obtained in dual 340 and 380 nm excitation mode and the average intensity of fluorescence in each region was used to estimate 340:380 ratios (R), reflecting [Ca^2+^]i. MetaMorph (Universal Imaging, Downington, PA) and SigmaPlot (Systat Software, San Jose, CA) scientific software were used for conversion and analysis of the acquired data. For quantitative measurement of changes in live cells for [Ca^2+^]i and oscillation frequency, the AutoFit function of PeakFit software (Systat Software, San Jose, CA) was used, with manual adjustments. The peak threshold was chosen empirically as ΔR≥0.05. Individual cells were considered responsive to estrogen treatment when they demonstrated increases in [Ca^2+^]i oscillation frequency of at least 0.25 calcium spikes per min (estimated during 10 min time interval) compared to the basal level, and delayed by no more than 10 min from the addition of any estrogen into the bath. Subsequent comparative analysis of cell responses was performed on responsive cells only for changes in intracellular Ca levels (ΔCa/ΔCa_0_; Figure 3D-F) and changes in (ΔCa) oscillation frequency (Figure 3G-I).

### Quantitative ERK phosphorylation and mER assays

We developed this assay to assess levels of activated ERKs 1 and 2 in fixed GH_3_/B6/F10 cells [72]. Cells were plated at 10,000 cells/well in poly-D-lysine-coated 96-well plates, and growth media were replaced the next day with DMEM containing 1% charcoal-stripped (4×) serum for 48 hr. Cells were then washed and treated with different estrogenic compounds in medium for 5 min, fixed with 2% paraformaldehyde/0.2% picric acid at 4°C for 48 hr, then permeabilized with PBS/2% BSA/0.1% Triton X-100 for 1 hr at RT. Cells were washed 3× with PBS and treated with primary Ab against pERKs (p-Thr202/Tyr204; 1:400 in PBS/1% BSA). After overnight incubation at 4°C, the cells were processed for signal development with the Vectastain ABC kit (Vector Labs, Burlingame, CA) as recommended by the company. Biotin-conjugated secondary Ab was used at a 1:300 dilution. Plates were incubated in the dark for 20 min at 37°C for the generation of alkaline phosphatase product (*para*-nitrophenol) and read at A_405 _in a model 1420 Wallace microplate reader (Perkin Elmer, Waltham, MA). The number of cells in each well was estimated by the crystal violet assay (see below) and used to normalize signals.

The membrane localization of ERs is based on preventing cell permeabilization by careful optimization of fixation and handling techniques for a given cell type. For measurements of mERs, the detergent permeabilization step (above) was left out and the status of the cells' permeabilization was measured by probing with Ab to clathrin (residing just under the plasma membrane); a low clathrin signal indicates intact cells [73]. Primary Abs were used at 1:1000 for ERα Ab, 1:2000 for ERβ Ab, 1:1000 for GPER Ab, and 1:400 for clathrin Ab.

### Prolactin assay

Cells (0.5-0.7 × 10^6^) were plated in poly-D-lysine-coated well of six-well plates. After serum deprivation in 1% charcoal-stripped (4×) serum for 48 h, this medium was removed and DMEM/0.1% BSA with hormone or the appropriate vehicle control (ethanol) was added. The cells were incubated for 2 min and centrifuged at 4°C (350 × *g*, 5 min), and the supernatant was collected and stored at -20°C. The PRL concentrations were determined using components of the rat PRL RIA kit from the National Institute of Diabetes and Digestive and Kidney Disease and the National Hormone and Pituitary Program (Baltimore, MD). Briefly, RIA buffer (80% PBS, 20% DMEM, 2% normal rabbit serum), 100 μL cold standard (rat PRL-RP-3) or unknown sample, rPRL-s-9 antiserum (final dilution of 1:437,500 in RIA buffer), and [^125^I]-rat-PRL (PerkinElmer, Wellesley, MA; 15,000 counts diluted in RIA buffer) were combined and incubated overnight at 4°C. Anti-rabbit IgG (Sigma) was added to a final dilution of 1:9, and the samples were incubated at RT for 2 hr. Then 1 mL of polyethylene glycol solution (1.2 M polyethylene glycol, 50 mM Tris, pH 8.6) was added and samples were incubated at RT for 15 min. After centrifugation at 4,000 × *g *for 10 min at 4°C, the supernatant was decanted and the pellet was counted in a Wizard 1470 Gamma Counter (PerkinElmer, Boston, MA). The PRL concentration was then normalized to the crystal violet (CV) values (estimates of the number of cells in each well). These measurements (n = 18) were done in 3 different experiments on different days using different passages of cells.

### Crystal violet assay

After being processed for the ERK, mER, and PRL assays, cells were washed 3× with PBS to remove media or *para*-nitrophenol and stained with 0.1% crystal violet solution for 30 min, then destained in deionized water. The dye was then released with 10% acetic acid, and the A_590 _signal of the extract was read in a model 1420 Wallace microplate reader [74]. The crystal violet assay has been shown previously to correlate well with other cell counting methods [75] and is used for its convenience of combining with our other assays in this study.

### Statistics

Data from [Ca^2+^]i, pERK, mER, and PRL studies were analyzed by one-way analysis of variance (ANOVA), followed by multiple comparisons vs. control group (Holm-Sidak method). Experiments were repeated at least 2-3 times using different passages of cells on different days. The Sigma Stat 3 program (Systat Software, Inc.) was used for all statistical analysis, and significance was accepted at p < 0.05.

## Results

We previously observed that initial estrogenic triggering of Ca influx into cells occurred at the same level for all effective estrogen concentrations -- an all-or-none response [15,17]. (This is different from the other graduated responses seen when escalating estrogen concentrations are presented to the same cells [19]). Therefore we chose only one active low concentration for all estrogenic compounds (10 pM) to represent a very low and common contamination level. We reasoned that such a low but effective concentration could best make the point regarding concern about prevalent environmental contamination levels.

We measured the portion of cells that respond with increases in Ca spiking (%, Figure 3A-C), the accumulated Ca levels in responding cells (ΔCa/ΔCa_0_, Figure 3D-F), and the Ca oscillation frequency (ΔCa oscillation, Figure 3G-I). All estrogens, both physiologic and nonphysiologic, caused substantial responses in all three types of Ca response measurements, as we have observed previously [15]. Only the percentage of cells responding to E_2 _was altered by combinations of physiologic estrogens with xenoestrogens. Attenuation was caused by combinations with 10 pM EP, PP, or NP, but not OP or BPA. Whether higher concentrations can affect this and other parameters of Ca signaling studied here will require further study.

**Figure 4:**
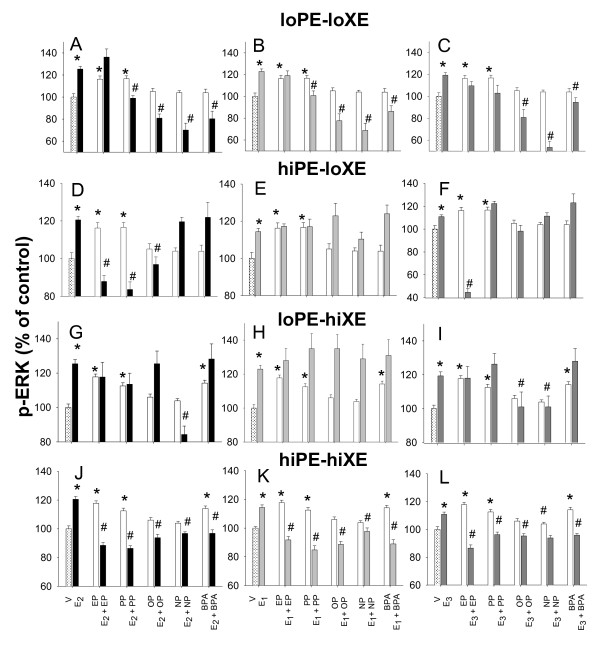
**Changes in pERK levels elicited by physiologic estrogens (PE) and xenoestrogens (XE), alone and in combination**. The levels of activated ERKs (pERK) were measured in cells treated with (A-C) 10 pM of the physiologic estrogens (E_2_, E_1_, or E_3 _) alone or in combinations with 10 pM of different alkylphenols (EP, PP, OP, NP, or BPA), row labeled lo PE - lo XE; (D-F) 1 nM of the physiologic estrogens alone or in combinations with 10 pM of different alkylphenols, row labeled hi PE - lo XE; (G-I) 10 pM of the physiologic estrogens alone or in combinations with 1 nM of different alkylphenols, row labeled lo PE - hi XE; (J-L) 1 nM of the physiologic estrogens alone or in combinations with 1 nM of different alkylphenols, row labeled hi PE - hi XE. * = p < 0.05 compared to vehicle (V) control cells. # = p < 0.05 compared to E_2_, E_1_, or E_3 _alone.

**Figure 5:**
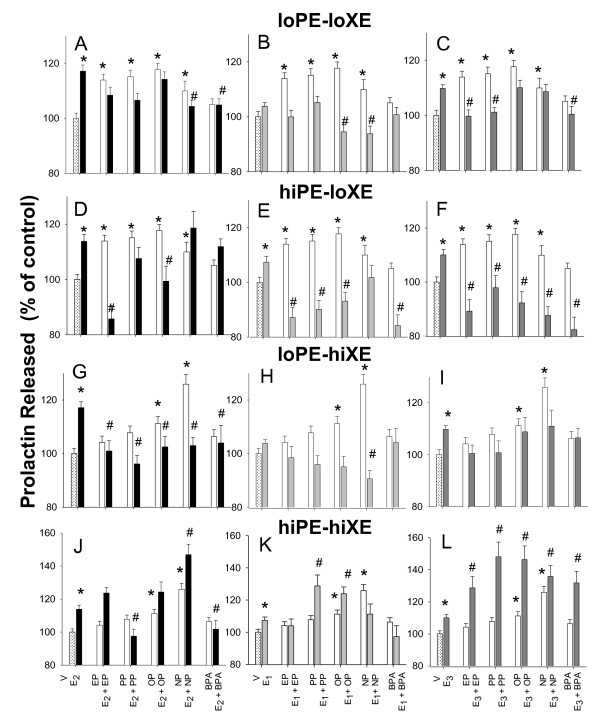
**Xenoestrogens alter the amount of prolactin released by physiologic estrogens**. The levels of prolactin (PRL) were measured in cells treated with (A-C) 10 pM of the physiologic estrogens (E_2_, E_1_, or E_3 _) alone or in combinations with 10 pM of different alkylphenols (EP, PP, OP, NP, or BPA), row labeled lo PE - lo XE; (D-F) 1 nM of the physiologic estrogens alone or in combinations with 10 pM of different alkylphenols, row labeled hi PE - lo XE; (G-I) 10 pM of the physiologic estrogens alone or in combinations with 1 nM of different alkylphenols, row labeled lo PE - hi XE; (J-L) 1 nM of the physiologic estrogens alone or in combinations with 1 nM of different alkylphenols, row labeled hi PE - hi XE. * = p < 0.05 compared to vehicle (V) control cells. # = p < 0.05 compared to E_2_, E_1_, or E_3 _alone.

Using higher through-put assays (in multi-well plates for the ERK and PRL studies), we tested multiple estrogen concentrations. Figures 4 and 5 show how combinations of two different endogenous estrogen concentrations with two different concentrations of each xenoestrogen (10 pM and 1 nM for all estrogens) can affect ERK activation and PRL release. These low concentrations of xenoestrogens correspond to ~20 ppb for the 1 nM concentration, and 200 ppt for the 10 pM concentration. They were chosen to represent high and low values of serum and urine levels reported in Americans [34]. For comparison, physiologic estrogens are present in these assays at ~27 ppb and 270 ppt concentrations.

In the ERK activation assays (Figure 4) both concentrations of all physiologic estrogens evoked responses. Only short-chain alkylphenols (EP and PP), and sometimes BPA activated ERK; (all showed increased activity, but not all instances were statistically significant). Xenoestrogens often blocked physiologic estrogen activation, especially when the higher concentrations of both estrogens were present (J-L). Demonstrating the non-monotonic behavior of xenoestrogen responses, low concentrations were sometimes more effective (disruptive of this response) than higher concentrations (compare A-F with G-L). The ability of BPA to disrupt depended upon whether it was balanced with an equal concentration of its paired physiologic estrogen (compare A-C and J-L, with D-I). In these concentration scenarios, xenoestrogens never significantly enhanced a physiologic estrogen response, as we have sometimes seen at other concentrations [76]. There are many instances where though a concentration of a long-chain alkylphenol or BPA was not active by itself, it could nevertheless inhibit the paired physiologic estrogen's response (seen in all panels except E, F, and H). In general, our data indicate that both high and low endogenous hormone levels are vulnerable to even extremely low xenoestrogen exposures, so both developing and adult females, and males, could be affected by these xenoestrogens.

In the PRL assays (Figure 5), both concentrations of all physiologic estrogens evoked responses, except that E_1 _did not always cause a significant change (see B and H). Alkylphenols always evoked PRL release at the lower (pM) concentration (panels A-F), but only the long-chain alkylphenols caused this response at the higher (nM) concentration, indicating a non-monotonic behavior of the short-chain alkylphenols. BPA did not cause a response on its own at either of these concentrations, though it has been observed to be active at other concentrations in this response [15,19].

Next we directed our attention to the ability of xenoestrogens to alter the release of PRL elicited by physiologic estrogens. Both short-chain and long-chain alkylphenols were able to block physiologic estrogen responses at either high or low concentrations, intermittently across this range of studies. Interestingly, when both xenoestogens and physiologic estrogens were at the higher (nM) concentration, the effect was more often an enhanced release of PRL (panels J-L). Though not active by itself at these concentrations, BPA could disrupt some physiologic estrogen actions across all paired compound concentrations, and was able to enhance release when its 1 nM concentration was paired with a 1 nM E_3 _concentration (panel L). Therefore, both of the tested endogenous hormone levels were again vulnerable to alteration.

While for the ERK activations, xenoestrogens always caused inhibition of the response, for the PRL release response, 1/3 of the time the effect was instead enhancement of the response (all at the adult endogenous estrogen levels in combination with nM xenoestrogens). The chain length of the alkylphenols had some influence, even if differently, on these combinatorial responses with physiologic estrogens, though they were not as pronounced as the previous structure-based effects we saw when these xenoestrogens were administered alone [1,15]. Overall, our stringent analysis by ANOVA did not show significance of some effects that would have been judged significant by a student's t-test. However, one can often see trends in the "non-significant" data that still support these conclusions.

**Figure 6:**
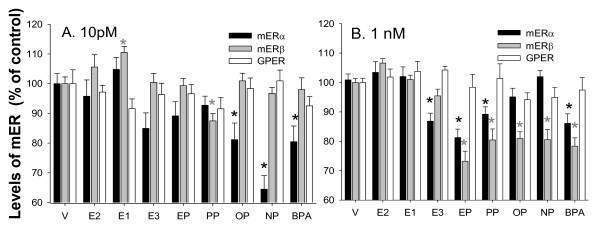
**Movement of mERs in or out of the membrane in response to different physiologic (E**_**2**_**, E**_**1**_**, or E**_**3 **_**) or environmental (EP, PP, OP, NP, or BPA) estrogens**. Levels were assessed by a quantitative plate assay measuring immunoreactive protein levels in the plasma membrane for ERα, ERβ, and GPER, after treatment with (A) 10 pM of estrogenic compounds or (B) 1 nM of estrogenic compounds for 5 min. *p < 0.05 compared to vehicle (V) control cells. #:p < 0.05 compared to E_2_, E_1_, or E_3 _alone.

Finally, we studied the effects of all of these estrogens on mER trafficking five minutes after hormone administration (Figure 6), choosing this time because it is unambiguously nongenomic, and because five-min responses were universally present for both ERK and Ca signaling pathways, and for the PRL secretion endpoint, for all classes of estrogens [1,15]. We had previously reported the lack of ERβ in our cell line [37], as values were not significantly different from controls (but did have wide errors of measurement). Perhaps due to assay improvements, cell line evolution, or change of Ab, we now see measurable amounts of ERβ in the membrane of these GH_3_/B6/F10 cells; whole cell ERβ had been reported by others in the parent GH_3 _cell line previously [77]. We detect a 57 kD band for ERβon an immunoblot at the same migration distance as ERβ isolated from LNCAP human prostate cancer cells (data not shown). Some xenoestrogen effects were seen at the 10 pM concentrations for most compounds; OP, NP and BPA all decreased mERα, and PP decreased mERβ. However, these effects were more prominent at the higher (1 nM) concentrations for all compounds. EP, PP, and BPA all decreased mERα in the membrane; all alkylphenols and BPA decreased mERβ in the membrane. It is interesting that physiologic estrogens also had some effects on these mERs. E_1_, ࿠increased levels of mERβ at the lower concentration and E_3 _decreased levels of mERα at the higher concentration. None of these estrogens significantly changed GPER levels.

To validate that our measured receptor antigens were in the membrane, and not the cell interior, we monitored our fixation conditions for absence from accidental plasma membrane disruption. To do this, we examined clathrin Ab recognition [73], expecting very low levels of this intracellular antigen in unpermeabilized cells. Here we saw a pNP/CV value of 0.035 ± 0.002 for clathrin in unpermeabilized cells compared to 0.16 ± 0.004 for permeabilized cells, a 4.6-fold higher value in permeabilized cells, as expected.

## Discussion

It is clear from our studies that combining xenoestrogens with different physiologic estrogens (E_1_, E_2_, and E_3_) alters responses driven by life-stage-specific levels of the endogenous estrogens. In some cases this caused inhibition of the physiologic response, while in others it caused enhancement. When xenoestrogens disrupt (either inhibit or enhance) estrogenic signaling, then development, menstrual cycling, pregnancy, or post-menopausal maintenance of estrogenic responses could all be altered. Life stage-inappropriate responses could terminate a pregnancy, cause infertility, cause birth defects, or alter pituitary-dependent regulation. Exacerbation of the known tumor-causing effects of estrogens could also be a toxic outcome. In all cases, the affected organism or population would function differently, and would likely be harmed.

Many tissues in both men and women have ERs. Besides the pituitary and reproductive organs, many other systems (eg. bone, brain, heart) could be negatively affected by the interference of xenoestrogen actions with physiologic estrogens. Functioning might be changed directly at the level of the ER-containing tissue, or via downstream activities (such as PRL released from the pituitary and then acting on other tissues). Many other tissues can be indirectly affected or need coordinate regulation by estrogenic actions because they are involved in supporting downstream reproductive functions. For example, pregnancy puts stress on bones due to weight gain, increases nutritional needs via the gut and associated metabolic organs, increases cardiovascular load, requires changes in behaviors, and must suspend some immune system responses to foreign (fetal) antigens. There are several tissues related to such changes upon which xenoestrogens have been reported to have effects at low concentrations [3,4,8-10,78]. Male reproductive and accessory glands also have ERs, and xenoestrogens have been shown to affect the size and function of these organs [79,80].

The levels of physiologic estrogens we have examined here represent those at two very vulnerable life stages. The low (10 pM) levels would be commensurate with an infant or young undeveloped male or female long before hormone levels rise at puberty. Certainly environmental estrogens could have an impact at this stage, when endogenous estrogens have not begun to rise to the high levels required for reproductive tissue development and function. The second level that we examined (1 nM) typifies cycling females and early pregnancy, and so would represent vulnerabilities to women during their reproductive years. The levels of xenoestrogens we studied represent those amounts expected to be present in the majority of Americans according to the values reported by the NHANES database [34,81] for human blood and urine, and are probably similar in inhabitants of other developed countries. Exposures to xenoestrogens, especially at the low doses that we study (10 pM-nM or 0.2-20 ppb), are therefore quite common as a result of present-day environmental exposures. We see evidence of non-monotonic dose-response behaviors throughout our study results, a pattern that is now recognized as typical of environmental and physiological estrogen actions via the nongenomic pathways. The hormesis effect [82] is somewhat evident here, where the more potent are estrogenic effects, the greater is the potential for inhibition of those effects at higher exposure levels, in this case exacerbated by the combination of estrogens. However, we can see these effects more prominently in other studies where more detailed dose responses were monitored [83]. Hormetic inhibitions are very common in hormonal responses of many kinds, and are thought by some to be safety mechanisms to prevent overstimulation. It appears that for the purposes of adding up the stimuli leading to signal integration at the level of the MAPKs [1], both physiologic and nonphysiologic estrogens contribute to the final count, perhaps pushing the total into the overstimulation range.

The mediators of these nongenomic responses are mERs, so it is important to understand if their levels in the membrane changed with these treatments. We had previously observed such movements of mERs in and out of the membrane in PC12 neuronal cells [13,14]; our findings there were consistent with the increased presence of mERα in the membrane as the primary mediator of estrogen-induced dopamine efflux from these cells via the dopamine transporter. mERβ and GPER acted to mediate inhibition of this response, and were consequently removed from the membrane under the influence of a variety of estrogens that enhanced dopamine effux. It is also possible that these receptors do not actually leave the membrane, but may be shifted to an inactive conformation that epitope-specific Abs to ERs no longer recognize. However, that is not likely with the Abs we used, because such conformational transformations affecting Ab recognition usually only occur in the hinge region epitopes of ERs [84,85], and here we used Abs recognizing the carboxy terminus. The physiologic estrogens E_1 _and E_3 _also altered the number of membrane ERβs and ERαs, respectively, so this may represent life stage-specific modulation of physiologic functions by rapid movement of receptors in and out of the membrane, a way for one endogenous hormone to influence the actions of others. Because alkylphenols and BPA dramatically downregulated mERα and β in the membrane, especially at the higher (nM) concentrations, it seems that this could be one of the primary mechanisms of their actions. Even though xenoestrogens did not affect GPER levels in the membrane in our study, several have been shown to bind to GPERs expressed by transfection in HEK293 cells [86]. Interestingly, E_3 _has recently been shown to be an antagonist at GPER, though it does not alter the presence of GPER in the membrane [87].

Trafficking actions would not only affect the signaling and functional responses to xenoestrogens at this rapid time point, but also have consequences for the subsequent actions of physiologic estrogens via these receptors. Where these receptors go, or when they return, is not addressed by our studies, but others have suggested that dynamic movement in and out of the membrane is part of the necessary cycling that supports estrogenic functions in a variety of tissues [88,89].

## Conclusions

By designing medium-throughput, quantitative assays to assess multiple nongenomic responses, we have been able to compare the potencies and efficacies of several structurally related xenoestrogens for important parameters of signaling, function, and receptor trafficking in pituitary cells. In contrast to their weak actions via genomic signaling pathways, these compounds are profoundly effective in disrupting nongenomic responses to multiple endogenous estrogens at concentrations that represent different life stages. These mechanistic explanations for the activities of xenoestrogens at low concentrations help explain their actions on the functions of exposed animals and humans both during development and in reproductive adulthood, and confirm our concern about continuing to allow such xenoestrogens to contaminate our environment.

## List of abbreviations

(EP): 4-*n*-ethylphenol; (NP): 4-*n*-nonylphenol; (OP): 4-*n*-octylphenol; (PP): 4-*n*-propylphenol; (Ab): antibody; (BPA): bisphenol A; (Ca): calcium; (E_2_): estradiol; (E_3_): estriol; (E_1_): estrone; (ER): estrogen receptor; (ERKs): extracellular-signal regulated kinases; (mERs): membrane estrogen receptors; (MAPKs): mitogen-activated protein kinases; (PRL): prolactin

## Competing interests

The authors declare that they have no competing interests.

## Authors' contributions

YJJ carried out the PRL, mER, and kinase experiments in these studies. MYK performed the imaging studies of intracellular Ca. All authors participated in the design and analyses of the studies, and wrote, read, and approved the final manuscript.

## References

[B1] Watson CS Jeng YJ Kochukov MY Nongenomic signaling pathways of estrogen toxicity Toxicol Sci 2010 115 1 11 10.1093/toxsci/kfp288 19955490 PMC2902922

[B2] Alonso-Magdalena P Laribi O Ropero AB Fuentes E Ripoll C Soria B Nadal A Low doses of bisphenol A and diethylstilbestrol impair Ca2+ signals in pancreatic alpha-cells through a nonclassical membrane estrogen receptor within intact islets of Langerhans Environ Health Perspect 2005 113 969 977 10.1289/ehp.8002 16079065 PMC1280335

[B3] Della SD Minder I Dessi-Fulgheri F Farabollini F Bisphenol-A exposure during pregnancy and lactation affects maternal behavior in rats Brain Res Bull 2005 65 255 260 10.1016/j.brainresbull.2004.11.017 15811589

[B4] Fujimoto T Kubo K Aou S Prenatal exposure to bisphenol A impairs sexual differentiation of exploratory behavior and increases depression-like behavior in rats Brain Res 2006 1068 49 55 10.1016/j.brainres.2005.11.028 16380096

[B5] Jones DC Miller GW The effects of environmental neurotoxicants on the dopaminergic system: A possible role in drug addiction Biochem Pharmacol 2008 76 569 581 10.1016/j.bcp.2008.05.010 18555207

[B6] Kabil A Silva E Kortenkamp A Estrogens and genomic instability in human breast cancer cells--involvement of Src/Raf/Erk signaling in micronucleus formation by estrogenic chemicals Carcinogenesis 2008 29 1862 1868 10.1093/carcin/bgn138 18544561

[B7] Kiguchi M Fujita S Oki H Shimizu N Cools AR Koshikawa N Behavioural characterisation of rats exposed neonatally to bisphenol-A: responses to a novel environment and to methylphenidate challenge in a putative model of attention-deficit hyperactivity disorder J Neural Transm 2008 115 1079 1085 10.1007/s00702-008-0044-5 18368283

[B8] Midoro-Horiuti T Tiwari R Watson CS Goldblum RM Maternal bisphenol a exposure promotes the development of experimental asthma in mouse pups Environ Health Perspect 2010 118 273 277 10.1289/ehp.0901259 20123615 PMC2831929

[B9] Munoz-de-Toro M Markey C Wadia PR Luque EH Rubin BS Sonnenschein C Soto AM Perinatal exposure to Bisphenol A alters peripubertal mammary gland development in mice Endocr 2005 10.1210/en.2005-0340 PMC2834307 15919749

[B10] Nadal A Alonso-Magdalena P Soriano S Quesada I Ropero AB The pancreatic beta-cell as a target of estrogens and xenoestrogens: Implications for blood glucose homeostasis and diabetes Mol Cell Endocrinol 2009 304 63 68 10.1016/j.mce.2009.02.016 19433249

[B11] Suzuki T Mizuo K Nakazawa H Funae Y Fushiki S Fukushima S Shirai T Narita M Prenatal and neonatal exposure to bisphenol-A enhances the central dopamine D1 receptor-mediated action in mice: enhancement of the methamphetamine-induced abuse state Neuroscience 2003 117 639 644 10.1016/S0306-4522(02)00935-1 12617968

[B12] Watson CS Jeng YJ Kochukov MY Nongenomic actions of estradiol compared with estrone and estriol in pituitary tumor cell signaling and proliferation FASEB J 2008 22 3328 3336 10.1096/fj.08-107672 18541692 PMC2518256

[B13] Alyea RA Watson CS Nongenomic mechanisms of physiological estrogen-mediated dopamine efflux BMC Neurosci 2009 10 59 10.1186/1471-2202-10-59 19531209 PMC2708169

[B14] Alyea RA Watson CS Differential regulation of dopamine transporter function and location by low concentrations of environmental estrogens and 17beta-estradiol Environ Health Perspect 2009 117 778 783 10.1289/ehp.0800026 19479021 PMC2685841

[B15] Kochukov MY Jeng YJ Watson CS Alkylphenol xenoestrogens with varying carbon chain lengths differentially and potently activate signaling and functional responses in GH_3_/B_6_/F10 somatomammotropes Env Health Perspect 2009 117 723 730 10.1289/ehp.0800182 PMC2685833 19479013

[B16] Jeng YJ Watson CS Proliferative and anti-proliferative effects of dietary levels of phytoestrogens in rat pituitary GH3/B6/F10 cells - the involvement of rapidly activated kinases and caspases BMC Cancer 2009 9 334 10.1186/1471-2407-9-334 19765307 PMC2755011

[B17] Jeng YJ Kochukov MY Watson CS Membrane estrogen receptor-alpha-mediated nongenomic actions of phytoestrogens in GH3/B6/F10 pituitary tumor cells J Mol Signal 2009 4 2 10.1186/1750-2187-4-2 19400946 PMC2679742

[B18] Bulayeva NN Watson CS Xenoestrogen-induced ERK-1 and ERK-2 activation via multiple membrane-initiated signaling pathways Environ Health Perspect 2004 112 1481 1487 10.1289/ehp.7175 15531431 PMC1325963

[B19] Wozniak AL Bulayeva NN Watson CS Xenoestrogens at picomolar to nanomolar concentrations trigger membrane estrogen receptor-alpha-mediated Ca2+ fluxes and prolactin release in GH3/B6 pituitary tumor cells Environ Health Perspect 2005 113 431 439 10.1289/ehp.7505 15811834 PMC1278483

[B20] Gaido KW Leonard LS Lovell S Gould JC Babai D Portier CJ McDonnell DP Evaluation of chemicals with endocrine modulating activity in a yeast-based steroid hormone receptor gene transcription assay Toxicol Appl Pharmacol 1997 143 205 212 10.1006/taap.1996.8069 9073609

[B21] Gutendorf B Westendorf J Comparison of an array of in vitro assays for the assessment of the estrogenic potential of natural and synthetic estrogens, phytoestrogens and xenoestrogens Toxicology 2001 166 79 89 10.1016/S0300-483X(01)00437-1 11518614

[B22] Steinmetz R Brown NG Allen DL Bigsby RM Benjonathan N The environmental estrogen bisphenol A stimulates prolactin release in vitro and in vivo Endocr 1997 138 1780 1786 10.1210/en.138.5.1780 9112368

[B23] Kloas W Lutz I Einspanier R Amphibians as a model to study endocrine disruptors: II. Estrogenic activity of environmental chemicals in vitro and in vivo Sci Total Environ 1999 225 59 68 10.1016/S0048-9697(99)80017-5 10028703

[B24] Sheeler CQ Dudley MW Khan SA Environmental estrogens induce transcriptionally active estrogen receptor dimers in yeast: activity potentiated by the coactivator RIP140 Environ Health Perspect 2000 108 97 103 10.2307/3454506 10656848 PMC1637889

[B25] Singleton DW Feng Y Chen Y Busch SJ Lee AV Puga A Khan SA Bisphenol-A and estradiol exert novel gene regulation in human MCF-7 derived breast cancer cells Mol Cell Endocrinol 2004 221 47 55 10.1016/j.mce.2004.04.010 15223131

[B26] Ikezuki Y Tsutsumi O Takai Y Kamei Y Taketani Y Determination of bisphenol A concentrations in human biological fluids reveals significant early prenatal exposure Human Reproduction 2002 17 2839 2841 10.1093/humrep/17.11.2839 12407035

[B27] Inoue K Yoshimura Y Makino T Nakazawa H Determination of 4-nonylphenol and 4-octylphenol in human blood samples by high-performance liquid chromatography with multi-electrode electrochemical coulometric-array detection Analyst 2000 125 1959 1961 10.1039/b006597h 11193082

[B28] Kawaguchi M Inoue K Sakui N Ito R Izumi S Makino T Okanouchi N Nakazawa H Stir bar sorptive extraction and thermal desorption-gas chromatography-mass spectrometry for the measurement of 4-nonylphenol and 4-tert-octylphenol in human biological samples J Chromatogr B Analyt Technol Biomed Life Sci 2004 799 119 125 10.1016/j.jchromb.2003.10.021 14659443

[B29] Takeuchi T Tsutsumi O Serum bisphenol A concentrations showed gender differences, possibly linked to androgen levels Biochem Biophys Res Commun 2002 291 76 78 10.1006/bbrc.2002.6407 11829464

[B30] Petrovic M Diaz A Ventura F Barcelo D Occurrence and removal of estrogenic short-chain ethoxy nonylphenolic compounds and their halogenated derivatives during drinking water production Environ Sci Technol 2003 37 4442 4448 10.1021/es034139w 14572098

[B31] Kolpin DW Furlong ET Meyer MT Thurman EM Zaugg SD Barber LB Buxton HT Pharmaceuticals, hormones, and other organic wastewater contaminants in U.S. streams, 1999-2000: a national reconnaissance Environ Sci Technol 2002 36 1202 1211 10.1021/es011055j 11944670

[B32] Bonefeld-Jorgensen EC Long M Hofmeister MV Vinggaard AM Endocrine-disrupting potential of bisphenol A, bisphenol A dimethacrylate, 4-n-nonylphenol, and 4-n-octylphenol in vitro: new data and a brief review Environ Health Perspect 2007 115 Suppl 1 69 76 10.1289/ehp.9368 18174953 PMC2174402

[B33] Calafat AM Kuklenyik Z Reidy JA Caudill SP Ekong J Needham LL Urinary concentrations of bisphenol A and 4-nonylphenol in a human reference population Environ Health Perspect 2005 113 391 395 10.1289/ehp.7534 15811827 PMC1278476

[B34] Lakind JS Naiman DQ Bisphenol A (BPA) daily intakes in the United States: estimates from the 2003-2004 NHANES urinary BPA data J Expo Sci Environ Epidemiol 2008 18 608 615 10.1038/jes.2008.20 18414515

[B35] Benoff S Modeling human sperm-egg interactions in vitro -- signal transduction pathways regulating the acrosome reaction Molecular Human Reproduction 1998 4 453 471 10.1093/molehr/4.5.453 9665632

[B36] Razandi M Pedram A Levin ER Plasma membrane estrogen receptors signal to antiapoptosis in breast cancer Mol Endocrinol 2000 14 1434 1447 10.1210/me.14.9.1434 10976921

[B37] Campbell CH Bulayeva N Brown DB Gametchu B Watson CS Regulation of the membrane estrogen receptor-alpha: role of cell density, serum, cell passage number, and estradiol FASEB J 2002 16 1917 1927 10.1096/fj.02-0182com 12468456 PMC1266276

[B38] Norfleet AM Thomas ML Gametchu B Watson CS Estrogen receptor-α detected on the plasma membrane of aldehyde-fixed GH3/B6/F10 rat pituitary cells by enzyme-linked immunocytochemistry Endocr 1999 140 3805 3814 10.1210/en.140.8.3805 10433242

[B39] Pappas TC Gametchu B Watson CS Membrane estrogen receptors identified by multiple antibody labeling and impeded-ligand binding FASEB J 1995 9 404 410 7896011 10.1096/fasebj.9.5.7896011

[B40] Acconcia F Ascenzi P Bocedi A Spisni E Tomasi V Trentalance A Visca P Marino M Palmitoylation-dependent estrogen receptor alpha membrane localization: regulation by 17beta-estradiol Mol Biol Cell 2005 16 231 237 10.1091/mbc.E04-07-0547 15496458 PMC539167

[B41] Marquez DC Pietras RJ Membrane-associated binding sites for estrogen contribute to growth regulation of human breast cancer cells Oncogene 2001 20 5420 5430 10.1038/sj.onc.1204729 11571639

[B42] Alyea RA Laurence SE Kim SH Katzenellenbogen BS Katzenellenbogen JA Watson CS The roles of membrane estrogen receptor subtypes in modulating dopamine transporters in PC-12 cells J Neurochem 2008 106 1525 1533 10.1111/j.1471-4159.2008.05491.x 18489713 PMC2574842

[B43] Razandi M Pedram A Greene GL Levin ER Cell membrane and nuclear estrogen receptors (ERs) originate from a single transcript: Studies of ER∀ and ERß expressed in chinese hamster ovary cells Mol Endocrinol 1999 13 307 319 10.1210/me.13.2.307 9973260

[B44] Thomas P Alyea R Pang Y Peyton C Dong J Berg AH Conserved estrogen binding and signaling functions of the G protein-coupled estrogen receptor 1 (GPER) in mammals and fish Steroids 2010 75 595 602 10.1016/j.steroids.2009.11.005 19931550 PMC2885585

[B45] Maggiolini M Picard D The unfolding stories of GPR30, a new membrane-bound estrogen receptor J Endocrinol 2010 204 105 114 10.1677/JOE-09-0242 19767412

[B46] Prossnitz ER Barton M Signaling, physiological functions and clinical relevance of the G protein-coupled estrogen receptor GPER Prostaglandins & Other Lipid Mediators 2009 89 89 97 10.1016/j.prostaglandins.2009.05.001 PMC2740807 19442754

[B47] Thomas P Dong J Binding and activation of the seven-transmembrane estrogen receptor GPR30 by environmental estrogens: a potential novel mechanism of endocrine disruption J Steroid Biochem Mol Biol 2006 102 175 179 10.1016/j.jsbmb.2006.09.017 17088055

[B48] Belcher SM Rapid signaling mechanisms of estrogens in the developing cerebellum Brain Res Rev 2008 57 481 492 10.1016/j.brainresrev.2007.07.020 17931703 PMC2322867

[B49] Zsarnovszky A Le HH Wang HS Belcher SM Ontogeny of rapid estrogen-mediated extracellular signal-regulated kinase signaling in the rat cerebellar cortex: potent nongenomic agonist and endocrine disrupting activity of the xenoestrogen bisphenol A Endocr 2005 146 5388 5396 10.1210/en.2005-0565 16123166

[B50] Narita S Goldblum RM Watson CS Brooks EG Estes DM Curran EM Midoro-Horiuti T Environmental estrogens induce mast cell degranulation and enhance IgE-mediated release of allergic mediators Environ Health Perspect 2007 115 48 52 10.1289/ehp.9378 17366818 PMC1797832

[B51] Wetherill YB Akingbemi BT Kanno J McLachlan JA Nadal A Sonnenschein C Watson CS Zoeller RT Belcher SM In vitro molecular mechanisms of bisphenol A action Reprod Toxicol 2007 24 178 198 10.1016/j.reprotox.2007.05.010 17628395

[B52] Jeng YJ Kochukov M Nauduri D Kaphalia BS Watson CS Subchronic exposure to phytoestrogens alone and in combination with diethylstilbestrol - pituitary tumor induction in Fischer 344 rats Nutr Metab (Lond) 2010 7 40 10.1186/1743-7075-7-40 20459739 PMC2881934

[B53] Gorski J Wendell D Gregg D Chun TY Estrogens and the genetic control of tumor growth. [Review] [23 refs] Progress in Clinical & Biological Research 1997 396 233 243 9108601

[B54] Zhu BT Liehr JG Quercetin increases the severity of estradiol-induced tumorigenesis in hamster kidney Toxicology & Applied Pharmacology 1994 125 149 158 10.1006/taap.1994.1059 8128490

[B55] Ho SM Tang WY Belmonte de FJ Prins GS Developmental exposure to estradiol and bisphenol A increases susceptibility to prostate carcinogenesis and epigenetically regulates phosphodiesterase type 4 variant 4 Cancer Res 2006 66 5624 5632 10.1158/0008-5472.CAN-06-0516 16740699 PMC2276876

[B56] Andrade AJ Grande SW Talsness CE Grote K Chahoud I A dose-response study following in utero and lactational exposure to di-(2-ethylhexyl)-phthalate (DEHP): non-monotonic dose-response and low dose effects on rat brain aromatase activity Toxicology 2006 227 185 192 10.1016/j.tox.2006.07.022 16949715

[B57] Alonso-Magdalena P Morimoto S Ripoll C Fuentes E Nadal A The estrogenic effect of bisphenol A disrupts pancreatic beta-cell function in vivo and induces insulin resistance Environ Health Perspect 2006 114 106 112 10.1289/ehp.8451 16393666 PMC1332664

[B58] Palanza P Gioiosa L vom Saal FS Parmigiani S Effects of developmental exposure to bisphenol A on brain and behavior in mice Environ Res 2008 108 150 157 10.1016/j.envres.2008.07.023 18949834

[B59] Warner KE Jenkins JJ Effects of 17alpha-ethinylestradiol and bisphenol A on vertebral development in the fathead minnow (Pimephales promelas) Environ Toxicol Chem 2007 26 732 737 10.1897/06-482R.1 17447558

[B60] Watson CS Norfleet AM Pappas TC Gametchu B Rapid actions of estrogens in GH_3_/B6 pituitiary tumor cells via a plasma membrane version of estrogen receptor-∀ Steroids 1999 64 5 13 10.1016/S0039-128X(98)00107-X 10323667

[B61] Vandenberg LN Maffini MV Sonnenschein C Rubin BS Soto AM Bisphenol-A and the great divide: a review of controversies in the field of endocrine disruption Endocr Rev 2009 30 75 95 10.1210/er.2008-0021 19074586 PMC2647705

[B62] Greenspan FS Gardner DG Greenspan FS, Gardner DG Appendix: Normal Hormone Reference Ranges Basic and Clinical Endocrinology 2004 7 New York: Lange Medical Books, McGraw Hill 925 926

[B63] Shenhav S Gemer O Volodarsky M Zohav E Segal S Midtrimester triple test levels in women with severe preeclampsia and HELLP syndrome Acta Obstet Gynecol Scand 2003 82 912 915 12956840 10.1034/j.1600-0412.2003.00250.x

[B64] Chard T Macintosh MC Screening for Down's syndrome J Perinat Med 1995 23 421 436 10.1515/jpme.1995.23.6.421 8904471

[B65] Meinhardt U Mullis PE The essential role of the aromatase/p450arom Semin Reprod Med 2002 20 277 284 10.1055/s-2002-35374 12428207

[B66] Jansson L Holmdahl R Enhancement of collagen-induced arthritis in female mice by estrogen receptor blockage Arthritis Rheum 2001 44 2168 2175 10.1002/1529-0131(200109)44:9<2168::AID-ART370>3.0.CO;2-2 11592382

[B67] Morley P Whitfield JF Vanderhyden BC Tsang BK Schwartz J A new, nongenomic estrogen action: The rapid release of intracellular calcium Endocr 1992 131 1305 1312 10.1210/en.131.3.1305 1505465

[B68] Selles J Polini N Alvarez C Massheimer V Novel action of estrone on vascular tissue: regulation of NOS and COX activity Steroids 2005 70 251 256 10.1016/j.steroids.2004.10.012 15784280

[B69] Chambliss KL Yuhanna IS Mineo C Liu P German Z Sherman TS Mendelsohn ME Anderson RG Shaul PW Estrogen receptor alpha and endothelial nitric oxide synthase are organized into a functional signaling module in caveolae Circ Res 2000 87 E44 E52 11090554 10.1161/01.res.87.11.e44

[B70] Zivadinovic D Watson CS Membrane estrogen receptor-alpha levels predict estrogen-induced ERK1/2 activation in MCF-7 cells Breast Cancer Res 2005 7 R130 R144 10.1186/bcr959 15642162 PMC1064105

[B71] Razandi M Oh P Pedram A Schnitzer J Levin ER ERs associate with and regulate the production of caveolin: Implications for signaling and cellular actions Mol Endocrinol 2002 16 100 115 10.1210/me.16.1.100 11773442

[B72] Bulayeva NN Gametchu B Watson CS Quantitative measurement of estrogen-induced ERK 1 and 2 activation via multiple membrane-initiated signaling pathways Steroids 2004 69 181 192 10.1016/j.steroids.2003.12.003 15072920 PMC1201430

[B73] Campbell CH Watson CS A comparison of membrane vs. intracellular estrogen receptor-alpha in GH(3)/B6 pituitary tumor cells using a quantitative plate immunoassay Steroids 2001 66 727 736 10.1016/S0039-128X(01)00106-4 11522334

[B74] Lottering ML Haag M Seegers JC Effects of 17β-estradiol metabolites on cell cycle events in MCF-7 cells Cancer Res 1992 52 5926 5932 1327520

[B75] Zivadinovic D Gametchu B Watson CS Membrane estrogen receptor-alpha levels in MCF-7 breast cancer cells predict cAMP and proliferation responses PMCID:15642158 Breast Cancer Res 2005 7 R101 R112 10.1186/bcr958 15642158 PMC1064104

[B76] Jeng YJ Watson CS Combinations of physiologic estrogens with xenoestrogens alter ERK phosphorylation profiles in rat pituitary cells Environ Health Perspect 2010 20870566 10.1289/ehp.1002512 PMC3018487

[B77] Mitchner NA Garlick C Steinmetz RW Ben-Jonathan N Differential regulation and action of estrogen receptors alpha and beta in GH3 cells Endocr 1999 140 2651 2658 10.1210/en.140.6.2651 10342855

[B78] Narita M Miyagawa K Mizuo K Yoshida T Suzuki T Prenatal and neonatal exposure to low-dose of bisphenol-A enhance the morphine-induced hyperlocomotion and rewarding effect Neurosci Lett 2006 402 249 252 10.1016/j.neulet.2006.04.014 16678967

[B79] vom Saal FS Cooke PS Buchanan DL Palanza P Thayer KA Nagel SC Parmigiani S Welshons WV A physiologically based approach to the study of bisphenol A and other estrogenic chemicals on the size of reproductive organs, daily sperm production, and behavior Toxicol Ind Health 1998 14 239 260 9460178 10.1177/074823379801400115

[B80] Welshons WV Nagel SC vom Saal FS Large effects from small exposures. III. Endocrine mechanisms mediating effects of bisphenol A at levels of human exposure Endocr 2006 147 S56 S69 10.1210/en.2005-1159 16690810

[B81] Stahlhut RW Welshons WV Swan SH Bisphenol A data in NHANES suggest longer than expected half-life, substantial nonfood exposure, or both Environ Health Perspect 2009 117 784 789 19479022 10.1289/ehp.0800376 PMC2685842

[B82] Weltje L vom Saal FS Oehlmann J Reproductive stimulation by low doses of xenoestrogens contrasts with the view of hormesis as an adaptive response Hum Exp Toxicol 2005 24 431 437 10.1191/0960327105ht551oa 16235731

[B83] Jeng YJ Watson CS Combinations of physiologic estrogens with xenoestrogens alter ERK phosphorylation profiles in rat pituitary cells Environ Health Perspect 2010 20870566 10.1289/ehp.1002512 PMC3018487

[B84] Watson CS Campbell CH Gametchu B Membrane estrogen receptors on rat pituitary tumor cells: Immunoidentification and responses to estradiol and xenoestrogens Experimental Physiology 1999 84 1013 1022 10.1111/j.1469-445X.1999.01903.x 10564698 PMC1931420

[B85] Norfleet AM Clarke C Gametchu B Watson CS Antibodies to the estrogen receptor-α modulate prolactin release from rat pituitary tumor cells through plasma membrane estrogen receptors FASEB J 2000 14 157 165 10627290 10.1096/fasebj.14.1.157 PMC1189731

[B86] Thomas P Dong J Binding and activation of the seven-transmembrane estrogen receptor GPR30 by environmental estrogens: a potential novel mechanism of endocrine disruption J Steroid Biochem Mol Biol 2006 102 175 179 10.1016/j.jsbmb.2006.09.017 17088055

[B87] Lappano R Rosano C De MP De Francesco EM Pezzi V Maggiolini M Estriol acts as a GPR30 antagonist in estrogen receptor-negative breast cancer cells Mol Cell Endocrinol 2010 320 162 170 10.1016/j.mce.2010.02.006 20138962

[B88] Song RX Barnes CJ Zhang Z Bao Y Kumar R Santen RJ The role of Shc and insulin-like growth factor 1 receptor in mediating the translocation of estrogen receptor alpha to the plasma membrane Proc Natl Acad Sci USA 2004 101 2076 2081 10.1073/pnas.0308334100 14764897 PMC357054

[B89] Pedram A Razandi M Sainson RC Kim JK Hughes CC Levin ER A conserved mechanism for steroid receptor translocation to the plasma membrane J Biol Chem 2007 282 22278 22288 10.1074/jbc.M611877200 17535799

